# A base editing resource for functional annotation of DNA repair variants in breast-derived cell models

**DOI:** 10.3389/fcell.2026.1714494

**Published:** 2026-03-26

**Authors:** Wardah Masud, Ludovica Russo, Vincent Chapdelaine-Trépanier, Samuel B. Hayward, Wei He, Marco Cirò, Ciro Mercurio, Han Xu, Alberto Ciccia, Raquel Cuella-Martin, Giuseppe Leuzzi

**Affiliations:** 1 Department of Human Genetics, McGill University, Montreal, QC, Canada; 2 Victor Philip Dahdaleh Institute of Genomic Medicine, McGill University, Montreal, QC, Canada; 3 IFOM ETS, The AIRC Institute of Molecular Oncology, Milan, Italy; 4 Department of Genetics and Development, Columbia University Irving Medical Center, New York, NY, United States; 5 Herbert Irving Comprehensive Cancer Center, Columbia University Irving Medical Center, New York, NY, United States; 6 Department of Epigenetics and Molecular Carcinogenesis, The University of Texas MD Anderson Cancer Center, Houston, TX, United States; 7 Department of Bioinformatics and Computational Biology, The University of Texas MD Anderson Cancer Center, Houston, TX, United States; 8 The Center for Cancer Epigenetics, The University of Texas MD Anderson Cancer Center, Houston, TX, United States; 9 Department of Biomedical Engineering, Columbia University, New York, NY, United States

**Keywords:** base editing screens, breast-derived models, DDR, functional genomics, precision oncology, VUS

## Abstract

**Background:**

The DNA damage response (DDR) safeguards genome integrity, and its disruption contributes to cancer development, therapy response, and resistance. Large-scale sequencing has identified thousands of DDR gene variants in tumors, but the functional consequences of most remain unclear, limiting their clinical interpretation and application.

**Results:**

We previously developed CRISPR-dependent base editing screens to functionally characterize DDR variants in breast-derived cell lines. Here, we extend this work to triple-negative breast cancer by performing a large-scale base editing screen in MDA-MB-231 cells. We assessed the impact on cellular fitness of ∼11,000 single-guide RNAs (sgRNAs) targeting the coding sequences of 27 DDR genes, primarily involved in homologous recombination (HR) and inter-strand crosslink repair (ICLR). The resulting dataset integrates mutation-associated effects with clinical annotations, enabling functional stratification of variants of uncertain significance.

**Conclusion:**

Combined with our previous datasets from MCF7 and MCF10A breast-derived cell lines, these results create a standardized, cross-comparable data that uncover both shared and context-specific genetic dependencies. Ultimately, we anticipate this resource will advance the functional interpretation of DDR variants, thereby facilitating the development of precision oncology approaches.

## Introduction

The DNA damage response (DDR) is a complex network of proteins that collectively safeguard genomic stability by detecting, signaling, and repairing a wide spectrum of DNA lesions (Zhou and Elledge, 2000). Defects in DDR components are frequently implicated in human genetic disorders, cancer development and progression, and resistance to cancer therapies (Jackson and Bartek, 2009; Ciccia and Elledge, 2010; Awwad et al., 2023). In particular, alterations in genes involved in homologous recombination (HR) and inter-strand crosslink repair (ICLR) have been closely linked to chromosomal instability, and heightened sensitivity to genotoxic therapies and DNA repair inhibitors (Bryant et al., 2005; Farmer et al., 2005; Konstantinopoulos et al., 2015; Groelly et al., 2023). As a result, mutations in DDR genes represent both biomarkers of therapeutic response and promising targets for precision oncology.

Large-scale cancer genome sequencing initiatives have identified thousands of single-nucleotide variants (SNVs) in DDR genes (Forbes et al., 2008; Cancer Genome Atlas Research et al., 2013; Consortium, 2020). While some of these variants have been classified as pathogenic or benign based on functional assays or genetic evidence, the vast majority remain uncharacterized and are therefore annotated as variants of uncertain significance (VUS) (Federici and Soddu, 2020). This represents a major bottleneck in clinical genetics, as current guidelines discourage the use of VUS information in clinical decision-making (Richards et al., 2015). Addressing this challenge requires scalable experimental frameworks capable of functionally annotating SNVs within their native genomic and regulatory contexts.

CRISPR-dependent base editing has emerged as a powerful platform for high-resolution variant characterization (Komor et al., 2016; Billon et al., 2017; Gaudelli et al., 2017). Unlike traditional CRISPR-Cas nucleases, base editors introduce targeted nucleotide substitutions without generating double-strand breaks, thereby minimizing cellular stress and enabling precise modeling of SNVs in endogenous loci. When integrated with high-throughput screening, base editing systematically interrogates thousands of candidate variants in parallel, enabling functional assessments at nucleotide resolution.

We and others have previously applied this approach to investigate the functional consequences of DDR gene variants in human cell models (Kweon et al., 2020; Cuella-Martin et al., 2021; Hanna et al., 2021; Huang et al., 2021; Lue and Liau, 2023). In our prior work, we performed base editing screens to assess the impact of DDR variants in the near-haploid HAP1 line and breast-derived systems, including the non-tumorigenic MCF10A cell line and the luminal breast cancer line MCF7 (Cuella-Martin et al., 2021). These studies demonstrated the ability of base editing to resolve the effect of individual variants on cell viability, DNA repair efficiency, and drug sensitivity, providing a functional framework to classify clinically relevant mutations (Cuella-Martin et al., 2021).

Triple-negative breast cancer (TNBC) is an aggressive and heterogeneous cancer subtype associated with genomic instability and poor prognosis (Derakhshan and Reis-Filho, 2022). Patients face limited treatment options, and conventional therapies often yield suboptimal outcomes (Foulkes et al., 2010). Importantly, genomic profiling has revealed that TNBC tumors frequently harbor mutations in DDR genes, particularly those involved in HR (Turner et al., 2004; Nik-Zainal et al., 2016; Polak et al., 2017). However, the impact of these variants remains largely unknown, limiting the ability to interpret their function and to exploit them as therapeutic targets.

In this study, we extend our prior CRISPR-dependent cytosine base editing platform (Cuella-Martin et al., 2021) to a TNBC model. We established a high-efficiency base editor–expressing MDA-MB-231 TNBC cell line and performed mutational tiling across 27 DDR genes mainly involved in HR and ICLR. Using a validated CRISPR-dependent base editing library (Cuella-Martin et al., 2021), we quantified the cellular fitness effects of individual SNVs, benchmarked variant classification against ClinVar annotation, and functionally characterized the impacts of VUSs. We further integrated normalized log2-fold change values (nLFC) across different breast-derived models to enable comparison between distinct genetic backgrounds. This work provides a resource to facilitate the interpretation of DDR variants and offers a foundation for exploring their implications in precision oncology.

## Materials and methods

### Cell lines and culture conditions

MDA-MB-231 cells were obtained from ATCC (https://www.atcc.org/en.aspx) and grown in DMEM High Glucose (#ECB7501L, Euroclone) supplemented with 10% (v/v) Fetal Bovine Serum (ECS5000L, Euroclone), 2 mM L-Glutamine (ECB3000D, Euroclone), 100 U/ml penicillin, and 100 μg/ml streptomycin (ECB3001L, Euroclone). MCF7 cells were grown DMEM High Glucose (#11965118, Thermo Fisher Scientific) supplemented with 10% (v/v) fetal bovine serum (#A5256701, Thermo Fisher Scientific), 100 U/ml penicillin, 100 μg/ml streptomycin and 0.25 μg/ml amphotericin B (#A5955, Sigma-Aldrich). MCF10A cells were grown in DMEM/Ham’s F12 medium (#10565, Thermo Fisher Scientific), supplemented with 5% (v/v) horse serum (#16050122, Thermo Fisher Scientific), 10 μg/mL insulin (Sigma-Aldrich), 0.5 μg/ml hydrocortisone (Sigma-Aldrich), 20 ng/ml human epidermal growth factor (PeproTech), and 100 U/ml penicillin, 100 μg/ml streptomycin and 0.25 μg/ml amphotericin B. All cells were cultured at 37 °C in a humidified atmosphere with 5% CO_2_.

### Plasmids

The BE3-FNLS-P2A-BlastR plasmid (Billon et al., 2017) was previously generated and used to establish MDA-MB-231 cells expressing the cytosine base editor. The pLenti-Guide-Puro vector (Addgene #52963) (Sanjana et al., 2014), containing either the sgRNA targeting the *AAVS1* locus or the pooled sgRNAs library, was also previously constructed (Cuella-Martin et al., 2021). LentiGuide fluorescent constructs used in two-color competitive growth assays were kindly provided by Daniel Durocher.

### Recombinant viral production and transduction

Recombinant lentiviruses were generated by co-transfecting helper packaging vectors together with lentiviral vectors into HEK293T cells using the TransIT-293 transfection reagent (Mirus) or using calcium-phosphate transfection. Virus-containing supernatants were collected 48–72 h after transfection and utilized at the indicated MOI to infect target cells in the presence of 8 μg/ml of polybrene. Forty-eight hours after viral addition, successfully infected cells were selected using puromycin (1 μg/ml), or blasticidin (10 μg/ml) for 3–5 days.

### Cell line generation

MCF7-BE3 and MCF10A-BE3 cell lines were generated previously (Cuella-Martin et al., 2021). To generate the MDA-MB-231-BE3 cell line, MDA-MB-231-GFP^+^ cells were transduced with lentivirus encoding the BE3-FNLS-P2A-BlastR base editor and selected with blasticidin. Three days after completion of drug selection, single-cell clones were isolated by fluorescence-activated cell sorting (FACS) into 96-well plates using a BD Influx Cell Sorter. Clones were expanded under continued blasticidin selection. Approximately 2 weeks post-sorting, eight individual clones exhibiting proliferation rates comparable to the parental pool were screened for base editing activity, and the clone with the highest editing efficiency (designated MDA-MB-231-BE3) was selected for downstream experiments.

### Analysis of base editing efficiency

Base editing efficiency was assessed by PCR amplification followed by BEAT analysis (https://hanlab.cc/beat/) (Xu et al., 2019), a computational method that deconvolutes Sanger sequencing chromatograms to estimate the frequency of base conversions at target positions. Briefly, cells were transduced with an sgRNA targeting the *AAVS1* safe harbor locus (sequence: 5′-GGG​GCC​ACT​AGG​GAC​AGG​AT-3′) as described above and harvested 7 days post-selection. Genomic DNA was extracted using QuickExtract DNA Extraction Solution (Lucigen,) according to the manufacturer’s instructions. The targeted *AAVS1* genomic region was PCR-amplified using the primers 5′-CTC​CTT​TCA​TTT​GGG​CAG​CTC-3’ (forward) and 5′-CTT​AGA​GGT​TCT​GGC​AAG​GAG​A-3’ (reverse), and the resulting amplicons were subjected to Sanger sequencing. Genomic DNA from non-transduced cells was used as a wild-type (WT) reference. Sequencing chromatograms were analyzed using the BEAT online tool to quantify single-base editing outcomes at the predicted target sites.

### Base editing screens

Base editing screens were performed in biological duplicates, maintaining a minimum library coverage of 500 cells per sgRNA throughout all stages. MDA-MB-231-BE3 cells were transduced with the lentiviral sgRNA library at a low multiplicity of infection (MOI <0.4) for 24 h. Selection was initiated by adding puromycin (1 μg/ml) for 48 h, after which cells were maintained in medium containing 0.5 μg/ml puromycin and 10 μg/ml blasticidin for the remainder of the screen. This point was designated as time 0 (T0). Cells were subcultured every 3 days and final collection was performed on day 38 (T38). Genomic DNA was extracted from T0 and T38 cell pellets using the Quick-DNA Midiprep Plus Kit (Zymo Research). Genome-integrated sgRNA sequences were PCR-amplified using Q5 High-Fidelity DNA Polymerase (New England Biolabs), and individual samples were barcoded as previously described (Cuella-Martin et al., 2021). The resulting libraries were sequenced (PE150) on an Illumina HiSeq sequencer by CD Genomics (https://www.cd-genomics.com).

### Variant annotation

Annotation of library variants was previously generated (Cuella-Martin et al., 2021) and includes both predicted mutational consequences (e.g., synonymous, missense, nonsense) and ClinVar-based clinical classifications (v. 2018-06-03). Briefly, all possible mutational outcomes for each sgRNA were generated by permuting the editable bases within the BE3 window (positions 13–18 from the PAM (Zafra et al., 2018)). Resulting base substitutions were translated into protein-level changes across all isoforms using the Variant Effect Predictor (Ensembl v93) and classified as synonymous, missense, nonsense, or splice variants. sgRNAs targeting exonic regions of predicted non-coding isoforms were grouped as putative non-coding, regardless of their mutational effect.

Each sgRNA was assigned to a single category corresponding to the most damaging mutation predicted in any isoform (e.g., if both a nonsense and a synonymous mutation were possible, it was classified as nonsense). Mutations in the first codon were categorized as nonsense, and sgRNAs without editable bases in the BE3 window were labeled as empty-window.

Predicted mutations were annotated against the ClinVar database using ANNOVAR (v2018Apr16; Wang et al., 2010) and grouped into three categories: (i) benign/likely benign, (ii) VUS (variants of uncertain significance or with conflicting interpretations), and (iii) pathogenic/likely pathogenic. When multiple variants were predicted for a single sgRNA, the clinical annotation of the most deleterious amino acid change was assigned. If all predicted outcomes were equally deleterious, clinical relevance was determined by the following hierarchy: benign/likely benign < absent in ClinVar (NA) < VUS < pathogenic/likely pathogenic.

### Sequencing data processing and screening quality control

Paired-end sequencing reads were trimmed at both the 5′ and 3′ ends using Cutadapt (v3.5) (Martin, 2011) to remove constant flanking sequences surrounding the sgRNA insert. Trimmed reads were quantified using the count function in MAGeCK (v0.5.9) to estimate sgRNA-level abundance (Li et al., 2014). Read counts were normalized using the median method in MAGeCK, and sgRNAs with zero counts in at least one replicate were removed before using the normalized counts for downstream analysis. To assess reproducibility between biological replicates, raw sgRNA counts were log2-transformed [log_2_ (counts +1)] and plotted as density-weighted scatterplots. Inter-replicate concordance was quantified using the Pearson correlation coefficient (r), calculated with scipy. stats.pearsonr (v1.14.1) in Python. For each replicate pair (i.e., T0_R1 vs. T0_R2 or T38_R1 vs. T38_R2), transformed values were visualized using Gaussian kernel density estimation (gaussian_kde) to highlight distribution patterns. Plots were annotated with r values, and median read counts were indicated with dashed lines. Finally, differential abundance analysis between T38 and T0 samples was performed using the test function in MAGeCK (v0.5.9). Normalized counts from MAGeCK were used as input to calculate log2-fold changes and corresponding p-values and FDR for each sgRNA.

Receiver operating characteristic (ROC) curves were generated using the roc_curve function from scikit-learn (v1.5.2) (Fabian Pedregosa et al., 2011). The area under the ROC curve (AUC) was calculated using the auc function, also from sklearn. metrics, providing a quantitative measure of classification performance. Higher AUC values indicate better discriminatory power between conditions. All plots were generated using matplotlib (v3.10.1) (Hunter, 2007).

### Normalized log2-fold change calculation and base editing screen dataset integration

Data were first normalized across conditions to account for differences in sequencing depth and potential batch effects. This normalization assumes most mutations do not produce a detectable phenotype, so each mutation’s read count ( $R_{i}$ ) is divided by the median read count of all mutations $median\left(R\right)$ in that condition:

$$R_{i}^{\prime }=\frac{R_{i}}{median\left(R\right)}$$

where $R_{i}^{\prime }$ is the normalized read count for the mutation i . Next, we calculated the log2-fold change (LFC) as phenotypic score $x_{i}$ by comparing $R_{i}^{\prime }$ to the normalized read counts ( $C_{i}^{\prime }$ ) in the D0 condition as follows:

$$x_{i}=\log_{2}\left(\frac{R_{i}^{\prime }+\varepsilon }{C_{i}^{\prime }+\varepsilon }\right)$$

Where ε is a small positive value to avoid large variation due to low read counts, and the default value was set to be 0.05. To facilitate comparison across different cell lines, we normalized the LFC data (nLFC) using the negative and positive controls in datasets (Meyers et al., 2017). The negative controls include sgRNAs targeting safe harboring *AAVS1* locus and non-targeting sgRNAs that do not alter the amino acid sequence, while iSTOP sgRNAs were used as positive controls. The nLFC values were calculated using the following formula:

$${nLFC}_{i}=\frac{x_{i}-\mu_{neg}}{\sigma_{ctrl}}$$

where $\mu_{neg}$ denotes the mean of the negative control distribution and $\sigma_{ctrl}$ denotes the standard deviation of both positive and negative controls. This normalization rescales mutation effects relative to the full dynamic range of the assay, enabling direct comparison across cell lines with differing baseline variability (Meyers et al., 2017). sgRNAs with an nLFC greater than 1 or less than −1 in at least one cell line were considered statistically significant and biologically relevant, corresponding approximately to one standard deviation beyond the neutral control distribution and reflecting a clear deviation from baseline variation across cell-line screens.

### Hierarchical clustering and enrichment analysis

Significant mutations and their corresponding phenotypic scores (nLFC across three cell lines) were organized into a matrix, with rows representing cell lines and columns representing individual mutations. Hierarchical clustering was performed on this matrix using Euclidean distance and Ward’s linkage to group mutations with similar functional profiles. To assess whether different mutation categories (P/LP, B/LB, or VUS, as annotated in ClinVar) were significantly enriched in the clusters identified from the screen, a hypergeometric test was applied. This test calculated the probability (p-value) of observing at least the given number of mutations of each category within a cluster, providing evidence for enrichment of clinically relevant variants.

### Two-color competitive growth assays

MCF7-BE3 and MCF10A-BE3 cells were transduced with lentiviral particles encoding either LentiGuide-NLS-mCherry-AAVS1 or LentiGuide-NLS-GFP–sgRNA-of-interest, whereas MDA-MB-231-BE3-GFP cells were transduced with lentiviral particles encoding either LentiGuide-NLS-AAVS1 or LentiGuide-NLS-mCherry–sgRNA-of-interest at a high multiplicity of infection (MOI >1). Twenty-four hours post-infection, puromycin was added at a final concentration of 1 μg/ml for 24 h (MDA-MB-231) or 2 μg/ml for 48 h (MCF10A and MCF7) to select for transduced cells. GFP- and mCherry-expressing populations were subsequently mixed at a 1:1 ratio and seeded into black, clear-bottom 96-well plates at a density of 5,000 cells per well for MCF10A experiments, 8,000 cells per well for MCF7 experiments, and 10,000 cells per well for MDA-MB-231 experiments (designated as day 0).

Cells were imaged on day 1 to assess initial plating ratios and subsequently on days 4, 8, 12, and 16. Image acquisition was performed using a Biotek Cytation C10 high-resolution Imaging Reader (Agilent, Santa Clara, CA) equipped with a ×10 objective for MCF10A and MCF7 cells, and an Operetta CLS High-Content Analysis System (PerkinElmer, Waltham, MA) equipped with a ×20 objective for MDA-MB-231 cells. Image segmentation and quantification of GFP- and mCherry-positive cells were performed using CellProfiler (Stirling et al., 2021) or Image Artist (Revvity) software. Each experiment was performed in at least two biological replicates, and each condition was assessed in technical replicates.

### Statistical analysis

Statistical analysis details for the different experiments are reported in figure legends or the methods section. In all cases: ns not significant; ∗ p-value <0.05; ∗∗ p-value <0.01; ∗∗∗ p-value <0.001; ∗∗∗∗ p-value <0.0001.

### Software

RStudio (v4.2.1), Python (v3), and GraphPad Prism (v9) were utilized in this study.

## Results

### CRISPR-dependent base editing screen to interrogate DDR variants in MDA-MB-231 cancer cells

In our prior work, we used CRISPR-dependent base editing screens to assess the functional impact of SNVs in DDR genes on cell fitness across three human cell lines: HAP1, MCF10A, and MCF7 (Cuella-Martin et al., 2021). To expand our variant-level functional analysis dataset and provide a broader resource for the scientific community, we extended our base editing screen to a TNBC model, which is characterized by genomic instability (Derakhshan and Reis-Filho, 2022) and limited therapeutic options (Foulkes et al., 2010).

**FIGURE 1:**
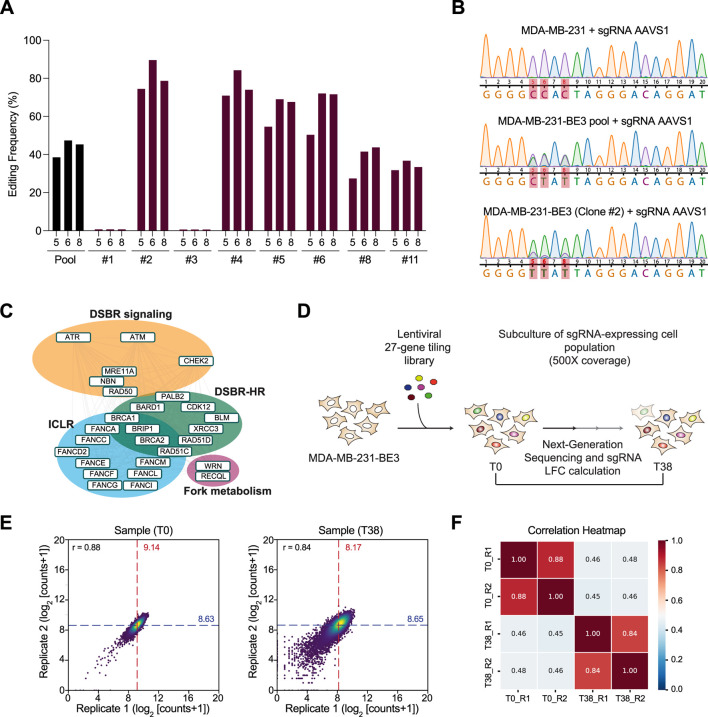
CRISPR-dependent base editing screen of DDR genes in MDA-MB-231 breast cancer cells. **(A)** Analysis of editing frequency at the *AAVS1* locus in MDA-MB-231 cells stably expressing the codon-optimized cytidine base editor (BE3), following transduction with an *AAVS1*-targeting sgRNA. Editing was measured on day 7 post-selection by Sanger sequencing and BEAT analysis. P: pooled population; #: individual clone. **(B)** Sanger sequencing chromatograms of the *AAVS1* locus corresponding to the analysis shown in **(A)**. Cytosines within the base editing window are highlighted in red. The top trace shows the wild-type sequence; the middle trace represents the edited sequence in the pooled population of MDA-MB-231 cells stably expressing BE3 (MDA-MB-231-BE3 pool); the bottom trace shows the edited sequence in the highest-efficiency clone (MDA-MB-231-BE3 Clone #2). In the result section referred to as MDA-MB-231-BE3. **(C)** Gene network targeted by the sgRNA library, organized by pathways, and their genetic and physical interactions (gray lines), according to the STRING database. Adapted from Cuella-Martin et al., *Cell*, 2021 (Cuella-Martin et al., 2021). **(D)** Schematic of the CRISPR-dependent base editing screen in MDA-MB-231 cells. Following selection of MDA-MB-231-BE3 cells transduced with the lentiviral sgRNA library, cells were cultured for 38 days (T38) prior to collection. The difference in sgRNA abundance between T0 and T38 was determined following next-generation sequencing. **(E,F)** Scatter plots displaying log2-transformed sgRNA read counts for the two biological replicates in MDA-MB-231 at baseline (T0) and endpoint (T38). The median of log2-transformed read counts (log_2_ [counts +1]) is indicated for each replicate **(E)**. The heatmap shows the Pearson correlation coefficients of sgRNA read coverage between replicates **(F)**.

To enable this analysis, we first generated MDA-MB-231 cells stably expressing a codon-optimized cytosine base editor (BE3-FNLS-P2A-BlastR). Individual cellular clones were subsequently isolated, and the clone exhibiting the highest activity (hereafter defined as MDA-MB-231-BE3) was selected for downstream experiments (Figures 1A,B). We then performed gene mutational tiling across DDR genes using our previously published lentiviral single-guide RNA (sgRNA) library targeting all NGG PAM sites within the coding regions of 27 genes primarily involved in HR and/or ICLR (Cuella-Martin et al., 2021) (Figure 1C). The library also includes sgRNAs designed to introduce premature stop codons in essential genes as positive controls for cell lethality (iSTOP controls) (Hart et al., 2015; Billon et al., 2017). As negative controls, we included non-targeting sgRNAs, sgRNAs targeting the *AAVS1* locus, and sgRNAs directed against essential genes lacking cytosines within the base editing window (empty–window controls) (Supplementary Table S1).

MDA-MB-231-BE3 cells were therefore transduced with the described lentiviral sgRNA library (∼11,000 sgRNAs) at approximately 500X representation and cultured for 38 days (Figure 1D). To quantify the impact of sgRNA-mediated base editing on cell fitness—defined as the ability of edited cells to survive and grow over time—we performed next-generation sequencing to measure sgRNA abundance at both the initial timepoint (T0) and the experimental endpoint (T38). Replicate analysis revealed high concordance of sgRNA read counts (Pearson’s r = 0.88 at T0; r = 0.84 at T38), indicating strong biological reproducibility of our screening approach and supporting its utility for mapping the effect of nucleotide variants in the MDA-MB-231 TNBC model at scale (Figures 1E,F).

### CRISPR-dependent base editing screen discriminates the mutational outcomes in DDR genes

**FIGURE 2:**
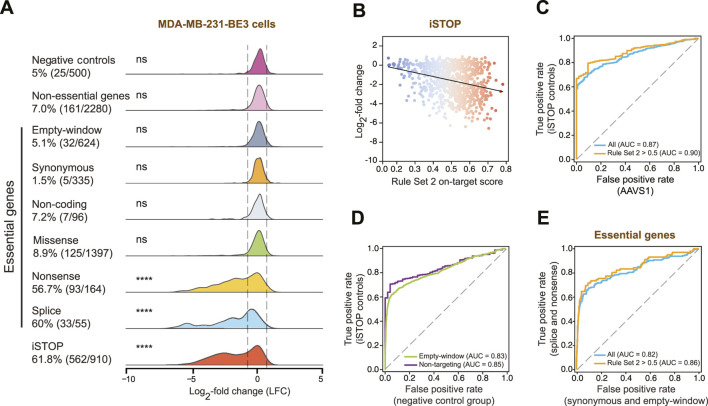
Performance of CRISPR-dependent base editing screens targeting DDR genes in MDA-MB-231 breast cancer cells. **(A)** Density plots of the log2-fold change (LFC) values distribution for sgRNAs predicted to generate the indicated SNVs in essential and non-essential genes in MDA-MB-231-BE3 cells (Supplementary Table S1). LFC density plots are also shown for iSTOP and negative control sgRNAs. Dotted lines, LFC for the bottom (LFC = −0.66) and the top (LFC = 0.73) 5% of negative controls. Statistical significance was assessed by Fisher’s exact test (ns not significant, ∗∗∗∗p-value <0.0001). **(B)** Dot plot of LFC values relative to Rule Set 2 on-target score for iSTOP control sgRNAs installed in MDA-MB-231 cells. **(C)** Receiver operating characteristic (ROC) curves generated based on the ranked LFC values for sgRNAs predicted to introduce iSTOP mutations (true positive) versus sgRNAs targeting *AAVS1* locus (false positive). The orange ROC curve represents only sgRNAs having Rule Set 2 on-target scores >0.5. Area under the curve (AUC) values are indicated in brackets. **(D)** ROC curves of true positives (sgRNAs introducing iSTOP controls) versus false positives (sgRNAs targeting empty-window, green curve; non-targeting sgRNAs, violet curve), based on the ranked LFC values. AUC values are shown in brackets. **(E)** ROC curves generated based on the ranked LFC values for sgRNAs predicted to introduce splice and nonsense mutations (true positive) versus synonymous and empty-window sgRNAs (false positives) in essential genes. The orange ROC curve represents only sgRNAs having Rule Set 2 on-target scores >0.5. AUC values are indicated in brackets.

To infer the impact of individual sgRNAs on cell fitness, we tracked changes in sgRNA abundance over time. Specifically, we compared sgRNA levels at the end of the experiment (T38) to those at the starting point (T0) and calculated, for each sgRNA, the log2-fold change (LFC) and its associated p-value. LFC reflects the relative enrichment or depletion of sgRNAs over time and quantifies the effect of the corresponding genetic perturbation on cellular fitness (Shalem et al., 2014; Wang et al., 2014; Hart et al., 2015). Then, we cataloged all possible DNA editing outcomes within the 6-nucleotide BE3 editing window (positions 13-18 from the PAM) (Zafra et al., 2018) and classified them into six distinct categories, ordered by predicted functional impact: (1) empty-window (no edit), (2) synonymous, (3) non-coding (targeting putative non-productive transcripts), (4) missense, (5) nonsense, and (6) splice. To evaluate the phenotypic consequences of these edits, we focused on controls and sgRNAs targeting the top 7 essential genes in our library (*ATR, BARD1, BRCA1, BRCA2, RAD51C, RAD51D, XRCC3*) and analyzed their LFC distributions across the above categories. In line with the expectations, analysis of the dropout profiles revealed a strong depletion of iSTOP control sgRNAs whose deleterious phenotypes increase with higher on-target efficiency values (Rule Set 2 on-target efficiency score) (Doench et al., 2016) (Figures 2A,B). sgRNAs predicted to generate nonsense or splice-disrupting mutations in the essential genes were significantly enriched among the most depleted (bottom 5% of negative controls), confirming their efficacy in generating loss-of-function alleles (Figure 2A). In contrast, empty-window, non-coding, and synonymous edits exhibited LFC distributions similar to negative controls, indicating minimal or no impact on cell fitness (Figure 2A), while missense mutations displayed intermediate LFC variability, reflecting their diverse functional effects in this cellular context.

To comprehensively evaluate screen performance, we performed multiple receiver operating characteristic (ROC) analyses. Comparing iSTOP sgRNAs to negative controls (*AAVS1*-targeting) yielded an area under the curve (AUC) of 0.87, which improved to 0.90 upon filtering sgRNAs with Rule Set 2 scores >0.5 (Figure 2C). Notably, ROC analysis confirmed that different negative controls (non-targeting, *AAVS1*-targeting, and empty-window sgRNAs) behaved indistinguishably, as expected (Figure 2D). Finally, ROC analysis comparing true positives (nonsense and splice-altering mutations) to false positives (empty-window and synonymous mutations) for sgRNAs targeting essential genes demonstrated strong discriminatory power (Figure 2E), with AUC values comparable to those observed in previous datasets across other cellular models. ROC analyses performed at the single-gene level likewise revealed robust discriminatory power for a subset of genes (Supplementary Figures S1, S2). Together, these results confirm the robustness of our base editing screening approach for large-scale functional analysis of SNVs in DDR genes and demonstrate its effective application in a TNBC model, thereby extending the relevance of our prior datasets to a distinct cellular model.

### Functional analysis of DDR variants of uncertain significance using base editing screens

**FIGURE 3:**
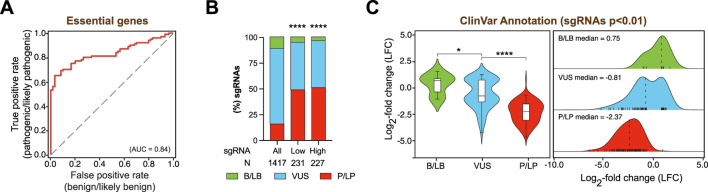
Functional classification of DDR variants using base editing screening in MDA-MB-231 breast cancer cells. **(A)** ROC curves generated based on the ranked LFC values for sgRNAs predicted to introduce pathogenic and likely pathogenic mutations (true positive) versus benign and likely benign sgRNAs (false positives) in essential genes. AUC value is indicated in brackets. **(B)** Stacked bar plots representing the distribution of sgRNAs predicted to introduce clinically relevant mutations in MDA-MB-231 cells, categorized as low-priority (LFC beyond top or bottom 5% of negative controls with p-value <0.05) or high-priority (LFC beyond top or bottom 5% of negative controls with p-value <0.01). Statistical significance was assessed by chi-square test relative to the full set of relevant sgRNAs (∗∗∗∗p-value <0.0001). **(C)** Violin plots with overlaid box plots (left) and corresponding kernel density estimates (right) illustrating the distribution of sgRNA LFC values across ClinVar-annotated variant categories, restricted to high-priority sgRNAs (LFC beyond top or bottom 5% of negative controls with p < 0.01). Dashed lines represent the median of LFC distributions in each different category. Statistical analysis was conducted using Mann-Whitney test (∗p-value <0.05, ∗∗∗∗p-value <0.0001).

Leveraging the ability of base editing screens to resolve the functional impact of DDR gene variants, we next evaluated whether our screening data in MDA-MB-231 cells could be used to infer the clinical relevance of variants of uncertain significance, as we previously demonstrated for MCF10A-BE3- and MCF7-BE3-associated datasets (Cuella-Martin et al., 2021). According to the ClinVar database (Landrum et al., 2014), the sgRNA library utilized in our screen included 157 sgRNAs predicted to introduce benign or likely benign (B/LB) mutations, 224 sgRNAs introducing pathogenic or likely pathogenic (P/LP) mutations, and 1,027 sgRNAs targeting VUS. To evaluate the capacity of our base editing approach to distinguish clinically relevant variants introduced by the library, we performed ROC analysis comparing true positives (P/LP) to false positives (B/LB) for sgRNAs targeting essential genes in MDA-MB-231-BE3 cells. This analysis showed strong separation between the two classes (AUC = 0.83), indicating that our screen could effectively distinguish pathogenic from benign variants (Figure 3A). Next, we examined the distribution of ClinVar-annotated sgRNAs across the entire library and among hits prioritized by increasing statistical significance (LFC beyond top or bottom 5% of negative controls with p-value <0.05, or p-value <0.01). We observed a clear enrichment of P/LP variants among these significant hits, consistent with an increased likelihood of functional impact at statistically significant confidence thresholds (Figure 3B). Finally, we focused on the high-priority (p-value <0.01) subset to evaluate the functional impact of clinically annotated sgRNAs. We found that sgRNAs introducing P/LP variants were significantly more deleterious to cell fitness than those introducing B/LB mutations, as expected (Figure 3C; Supplementary Figure S3). Notably, analysis of the LFCs associated with VUS-targeting sgRNAs revealed a bimodal distribution, indicating that a subset of VUS exhibits pathogenic-like behavior, while others resemble benign variants (Figure 3C). These findings support the utility of base editing screens as a functional classifier for clinically relevant variants of uncertain significance and extend our prior observations in MCF10A and MCF7 cells to the TNBC MDA-MB-231 model.

### Cross-cell line comparisons reveal context-specific effects of DDR variants

**FIGURE 4:**
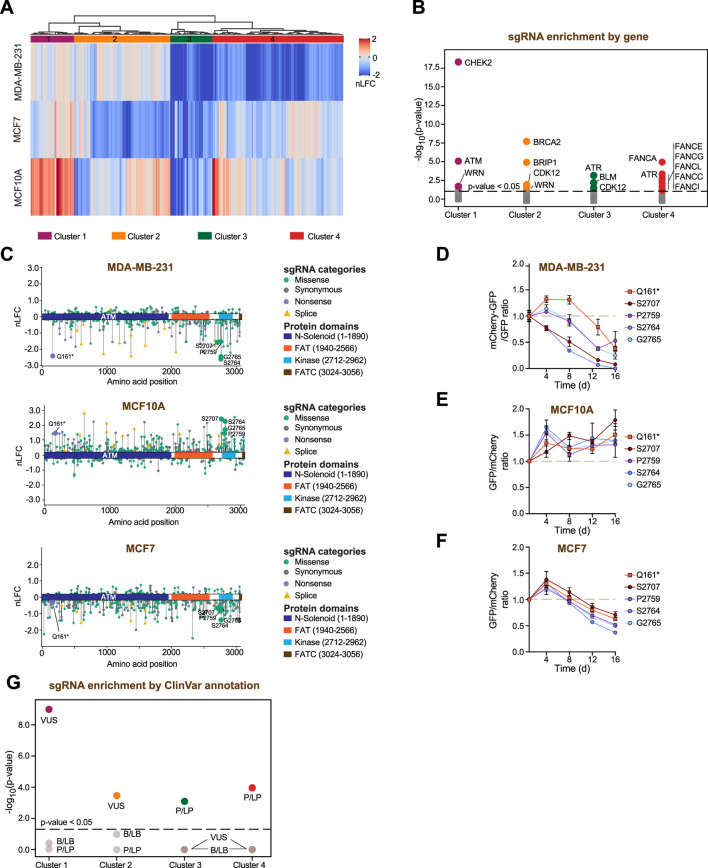
Integration of base editing screens across cellular models. **(A)** Heatmap showing the nLFC values for sgRNAs across three different breast-derived cellular models (MCF10A, MCF7, and MDA-MB-231). Clustering analysis was performed using Euclidean distance and Ward’s method. See Supplementary Table S1. **(B)** Enrichment analysis of sgRNA-associated variants targeting specific genes within the groups defined by the cluster analysis in **(A)**. The dotted line represents a p-value threshold of 0.05. See Supplementary Table S1. **(C)** Lollipop plots of ATM sgRNAs, with their nLFC values mapped to the canonical ATM protein isoform in three different breast-derived cellular models (MCF10A, MCF7, and MDA-MB-231). Residues validated in two-color competitive growth assays are highlighted next to their corresponding lollipops. **(D)** Competitive growth assay in MDA-MB-231-BE3-GFP cells. Data represent the sgRNA-of-interest-mCherry/*AAVS1* ratio normalized to the day 1 time point. Mean ± s. e.m. for n = 3. **(E,F)** Competitive growth assay in MCF10A-BE3 **(E)** or MCF7-BE3 **(F)** cells. Data represent the sgRNA-of-interest-GFP/*AAVS1*-mCherry ratio normalized to the day 1 time point and to the corresponding *AAVS1*-GFP/*AAVS1*-mCherry ratio at each experimental time point. Mean ± s. e.m. for n = 2–3. **(G)** Enrichment analysis of sgRNA-associated variants classified by ClinVar within the groups defined by the cluster analysis in **(A)**. The dotted line represents a p-value threshold of 0.05.

To facilitate the interrogation of DDR base editing screening datasets, we harmonized data from the MDA-MB-231 screen with our previously published datasets (Cuella-Martin et al., 2021) by computing normalized log2-fold change (nLFC) scores for each sgRNA based on changes in abundance between T0 and the experimental endpoint (Figure 4A; Supplementary Table S1). This normalization enables consistent cross-cell line comparisons and supports integrative analyses of the functional impact of DDR gene variants across diverse cellular contexts.

Interestingly, we observed that a subset of variants affects cell fitness in a cell-type–dependent manner. Hierarchical clustering of the integrated datasets revealed distinct modules of variants displaying differential effects across breast cancer cell lines and non-tumorigenic cells, suggesting context-specific dependencies (Figure 4A). To determine whether these variant-level clusters reflected underlying gene-specific requirements, we performed an enrichment analysis assessing whether sgRNAs targeting individual DDR genes were significantly overrepresented within each cluster (Figure 4B; Supplementary Table S1). This analysis revealed clear gene- and pathway-level associations. Specifically, sgRNAs targeting components of the ATM–CHEK2 signaling axis, key mediators of DNA damage checkpoint signaling (Zhou and Elledge, 2000), were significantly enriched in a cluster characterized by increased fitness in MCF10A cells and reduced fitness in both MCF7 and MDA-MB-231 cells (Figure 4B, Cluster 1). In contrast, sgRNAs targeting core homologous recombination genes, including BRCA2 and BRIP1, were overrepresented in a cluster marked by pronounced impairment of cell fitness predominantly in MCF7 cells (Figure 4B, Cluster 2). Additionally, sgRNAs targeting genes of the Fanconi anemia pathway were specifically enriched in a cluster characterized by reduced fitness primarily in MDA-MB-231 cells (Figure 4B, Cluster 4), whereas sgRNAs targeting ATR were enriched in a cluster associated with strong fitness defects across all breast-derived models (Figure 4B, Cluster 3). Together, these findings further highlight context-biased dependencies within the DNA damage response network.

To experimentally test these context-dependent effects, we focused on sgRNA-induced variants targeting ATM, which grouped within Cluster 1 and exhibited divergent fitness effects across the three breast-derived models (Figure 4B; Supplementary Table S1). Specifically, these variants were associated with increased fitness in MCF10A cells and reduced fitness in MDA-MB-231 and MCF7 cells, with a milder effect observed in MCF7 (Figure 4C). We selected a set of representative sgRNAs introducing distinct classes of mutations, including one sgRNA generating a nonsense mutation (Q161*), one sgRNA targeting the S2707 residue predicted to be edited to phenylalanine, and three sgRNAs targeting residues within the kinase domain (P2759, S2764, and G2765), predicted to be edited to serine/phenylalanine/leucine, asparagine, and aspartate/asparagine/serine, respectively (Figure 4C). The fitness effects induced by these sgRNAs were confirmed using a two-color competition assay performed in red- or green-fluorescently labeled MDA-MB-231, MCF10A, and MCF7 cell populations stably expressing the BE3 construct and either *AAVS1* control or *ATM*-targeting sgRNAs (Figures 4D–F). Together, these results demonstrate that dataset harmonization supports cross-cell line comparisons that capture biologically meaningful, context-dependent cellular fitness effects of DDR variants.

We next examined the relationship between the clusters of the integrated datasets (Figure 4A) and ClinVar annotations. Enrichment analysis revealed that the distribution of variants within clusters was significantly associated with P/LP mutations and VUS, but not with benign categories (Figure 4G). This observation suggests that the functional impact of DDR variants captured in our screens aligns with existing clinical annotations, while also providing experimental evidence to help refine the interpretation of VUS in a context-dependent manner.

## Discussion

In this study, we describe a CRISPR-dependent base editing dataset in which changes in cellular fitness relative to internal controls serve as the functional readout to interrogate the impact of single-nucleotide variants across DDR genes in the TNBC model MDA-MB-231. Moreover, we integrate the newly generated dataset with our previously published screens performed in MCF7 and MCF10A cell lines (Cuella-Martin et al., 2021). By generating and harmonizing variant-level data across these related but biologically distinct contexts, we provide a standardized and comparable resource to explore how DDR gene variants affect cell fitness within breast tissue–derived backgrounds. This integration is particularly valuable because it allows researchers to disentangle cell-type–specific effects from generalizable features, while maintaining direct relevance to breast cancer biology.

A key feature of this dataset is the use of cytosine base editing to generate precise nucleotide substitutions at scale (Komor et al., 2016; Gaudelli et al., 2017), which allowed us to capture both loss-of-function and more subtle missense outcomes. The dataset includes comprehensive quality control metrics, detailed annotation of mutational outcomes, and nLFC calculations that facilitate comparison across cellular backgrounds. Given the nature of the screening design, the dataset is not annotated using the ACMG/AMP framework for clinical variant interpretation (Brnich et al., 2019; Tavtigian et al., 2020); instead it integrates ClinVar annotations (Landrum et al., 2014), providing users with a clinically relevant reference point to contextualize variant-level functional data. Specifically, variants annotated as pathogenic, benign, or of uncertain significance in ClinVar are complemented here by a quantitative cellular fitness readout under unperturbed growth conditions. Our results indicate that the screen can distinguish pathogenic from benign variants and highlight subsets of VUS with measurable functional effects. This functional information does not directly inform therapeutic response or treatment sensitivity, but provides experimental context that can be integrated with future drug-based screens, genetic interaction studies, or clinical outcome data. Accordingly, while the dataset itself is not intended to provide clinical reclassification of VUS, it represents a valuable experimental framework that can inform future studies and computational approaches aimed at variant interpretation.

The harmonization of datasets across three mammary-derived cell lines further enhances reusability, as it allows researchers to investigate genetic background–specific effects of individual variants. While MCF10A offers a non-tumorigenic baseline, MCF7 represents a luminal breast cancer model, and MDA-MB-231 captures the biology of aggressive TNBC. Such diversity is particularly relevant for DDR pathways, where the cellular and chromatin context can strongly influence DNA repair activity, therapeutic responses, and overall cellular fitness (Aymard et al., 2014; Groelly et al., 2023; Vergara et al., 2024). By enabling side-by-side functional comparisons across these models, our dataset provides an experimental basis to investigate how cellular context shapes the functional consequences of DDR variants. We anticipate that the dataset will be useful not only for those studying DDR biology but also for groups interested in benchmarking computational predictors of variant function, training machine learning models for variant classification, and integrating experimental data with clinical or population-based sequencing studies.

As with any large-scale functional screen, some limitations should be considered. Base editing outcomes are constrained by the presence of editable bases within the sgRNA editing window and by PAM requirements, which limit the available target sites (Komor et al., 2016). Additionally, the efficiency of generating specific nucleotide changes can vary due to factors like GC content and chromatin structure, which can influence accessibility and editing precision (Komor et al., 2016). Moreover, the phenotypic readout of cell fitness, while robust and scalable, primarily reflects one dimension of DDR variant function. Accordingly, additional assays, such as drug-based perturbations, DNA repair–specific readouts, replication stress assays, or immune signaling measurements, will be required to extend the functional scope of this dataset. Finally, while we provide nLFC scores across cell lines, further integration with MAVE database (Rubin et al., 2025) and orthogonal datasets (e.g., CRISPR knockout screens, proteomics, patient-derived data) will broaden the interpretability and impact of these DDR variants.

## Conclusion

In conclusion, this dataset provides a robust and accessible resource for exploring the functional consequences of DDR gene variants in a TNBC model. By integrating these data with our previously developed online database (https://www.ciccialab-database.com/ddr-variants), we expand the availability of variant functional annotations in a format designed for broad reuse and interrogation. Through public accessibility and cross-model harmonization, this resource aims to support diverse applications—from computational method development to experimental validation—and to contribute to the broader community effort of improving the interpretation of genetic variants through open data sharing.

## Data Availability

The datasets presented in this study can be found in online repositories. The names of the repository/repositories and accession number(s) can be found in the article/Supplementary Material (https://www.ciccialab-database.com/ddr-variants).

## Supplementary material

SUPPLEMENTARY TABLE S1 The table contains raw and normalized read counts from the CRISPR-dependent base editing screen performed in MDA-MB-231 breast cancer cells (related to Figure 1). The table also reports log2-fold change values, p-values, and FDRs for each sgRNA calculated with MAGeCK by comparing timepoint T38 versus T0 in MDA-MB-231 cells. In addition, nLFC values, clustering analysis, enrichment analysis and ClinVar annotations associated with CRISPR-mediated base editing screens in MCF10A, MCF7 and MDA-MB-231 are provided.

SUPPLEMENTARY FIGURE S1 Receiver operating characteristic (ROC) curves generated based on the ranked LFC values for sgRNAs predicted to introduce splice and nonsense mutations (true positive) versus synonymous and empty-window sgRNAs (false positives) in the indicated genes.

SUPPLEMENTARY FIGURE S2 Receiver operating characteristic (ROC) curves generated based on the ranked LFC values for sgRNAs predicted to introduce splice and nonsense mutations (true positive) versus synonymous and empty-window sgRNAs (false positives) in the indicated genes.

SUPPLEMENTARY FIGURE S3 Box and scatter plots illustrating the distribution of sgRNA LFC values for each gene across ClinVar-annotated variant categories: B/LB (benign/likely benign), VUS (variant of uncertain significance), P/LP (pathogenic/likely pathogenic), and NA (variants without ClinVar annotation). Statistical analysis was conducted using Mann-Whitney test (ns, not significant, ∗p-value < 0.05, ∗∗p-value < 0.01, ∗∗∗p-value < 0.001).
