# Supplementary figures and images for: A base editing resource for functional annotation of DNA repair variants in breast-derived cell models
Source: Front Cell Dev Biol. 2026 Mar 26;14:1714494. doi: 10.3389/fcell.2026.1714494 (PMC13062485; doi:10.3389/fcell.2026.1714494)

A

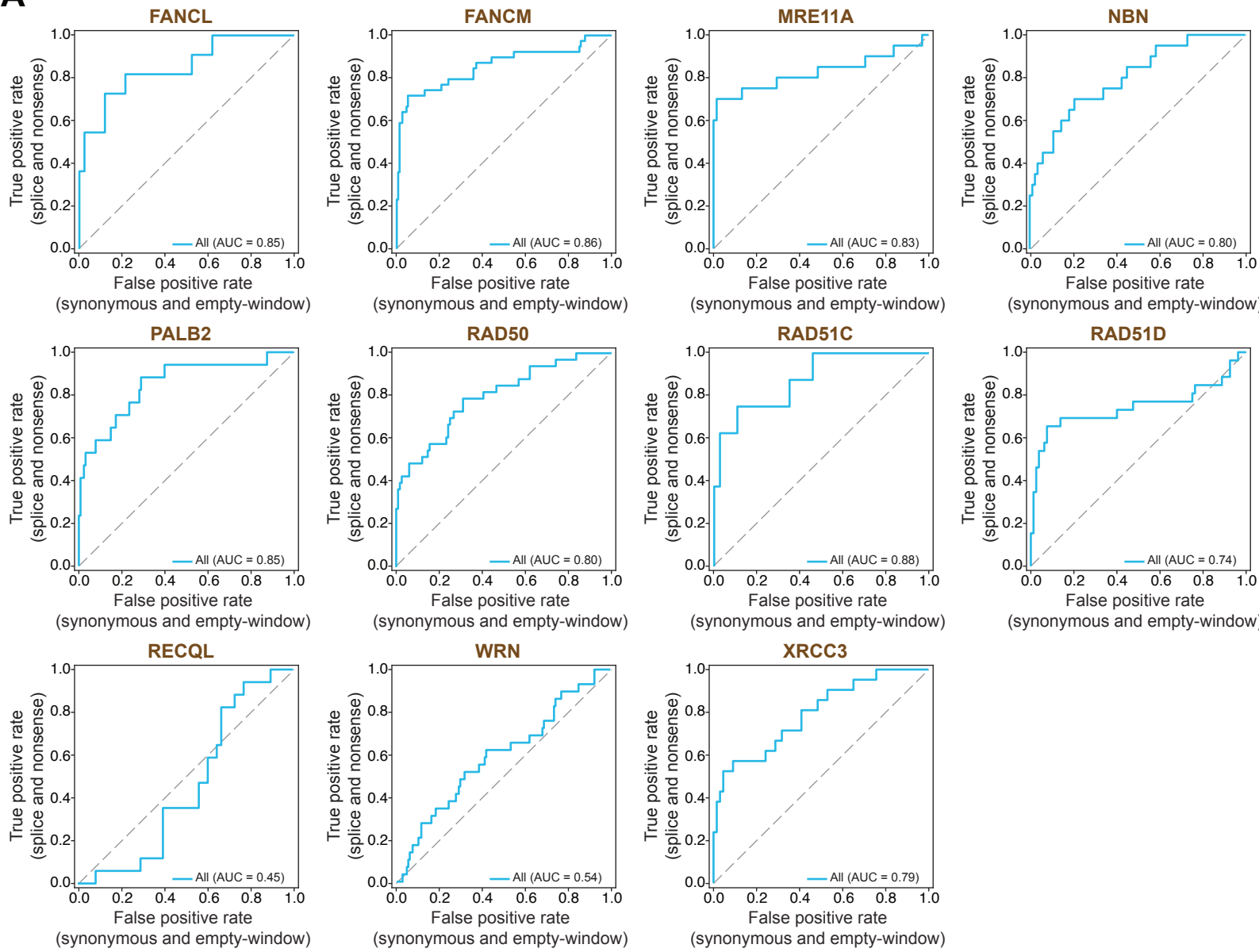

Supplement: Supplementary file 1 [file Image2.pdf]

Masud and Russo *et al.* Supplementary Figure 3

A

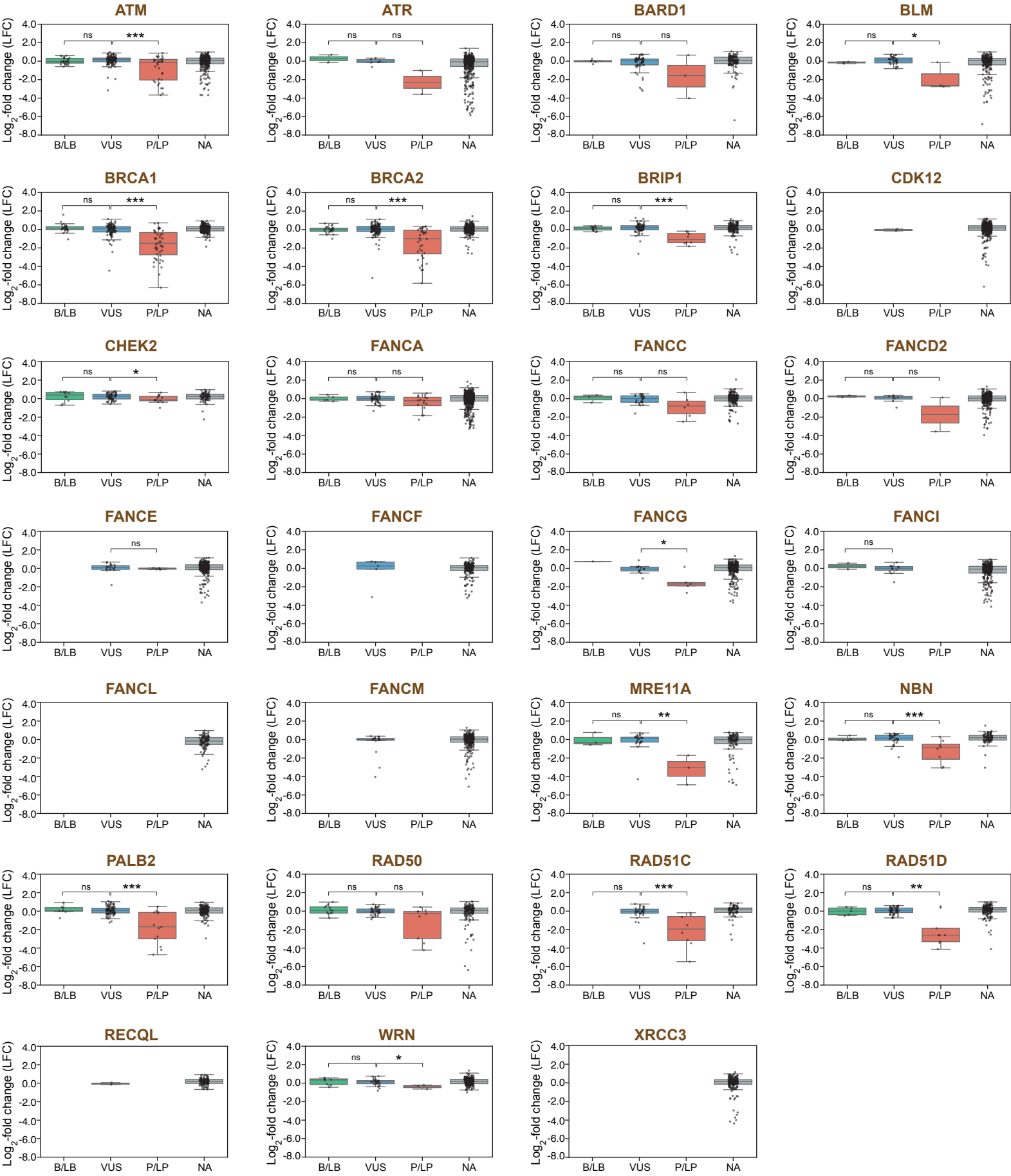

Supplement: Supplementary file 2 [file Image3.pdf]

A

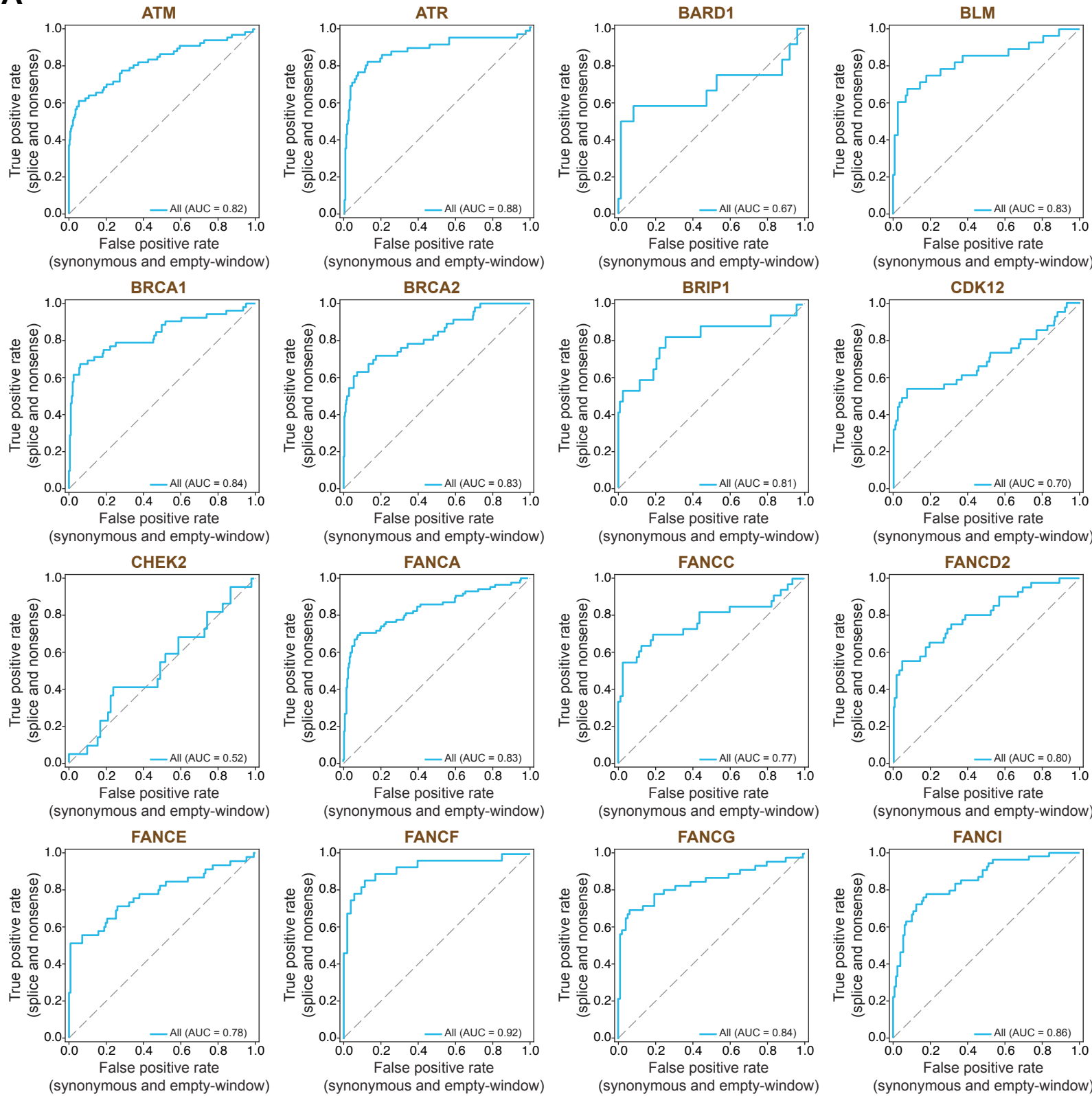

Supplement: Supplementary file 4 [file Image1.pdf]
